# Supplementary material for: Collation Efficiency of Poly(Vinyl Alcohol) and Alginate Membranes with Iron-Based Magnetic Organic/Inorganic Fillers in Pervaporative Dehydration of Ethanol
Source: Materials (Basel). 2020 Sep 18;13(18):4152. doi: 10.3390/ma13184152 (PMC7560291; doi:10.3390/ma13184152)
Supplement: Supplementary file 1 [file materials-13-04152-s001.pdf]

materials

Supporting Information

Collation Efficiency of Poly(Vinyl Alcohol) and Alginate Membranes with Iron-Based Magnetic Organic/Inorganic Fillers in Pervaporative Dehydration of Ethanol

Gabriela Dudek [1,*], Roman Turczyn [1] and David Djurado [2]

1 Department of Physical Chemistry and Technology of Polymers, Faculty of Chemistry, Silesian University of Technology, Strzody 9, 44-100 Gliwice, Poland; roman.turczyn@polsl.pl
2 Systèmes Moléculaires et nanoMatériaux pour l'Energie et la Santé (SyMMES), IRIG, CNRS, CEA Grenoble, Université Grenoble Alpes, 17 rue des Martyrs, 38054 Grenoble, France; david.djurado@cea.fr
* Correspondence: gmdudek@polsl.pl

Received: 18 August 2020; Accepted: 15 September 2020; Published: date

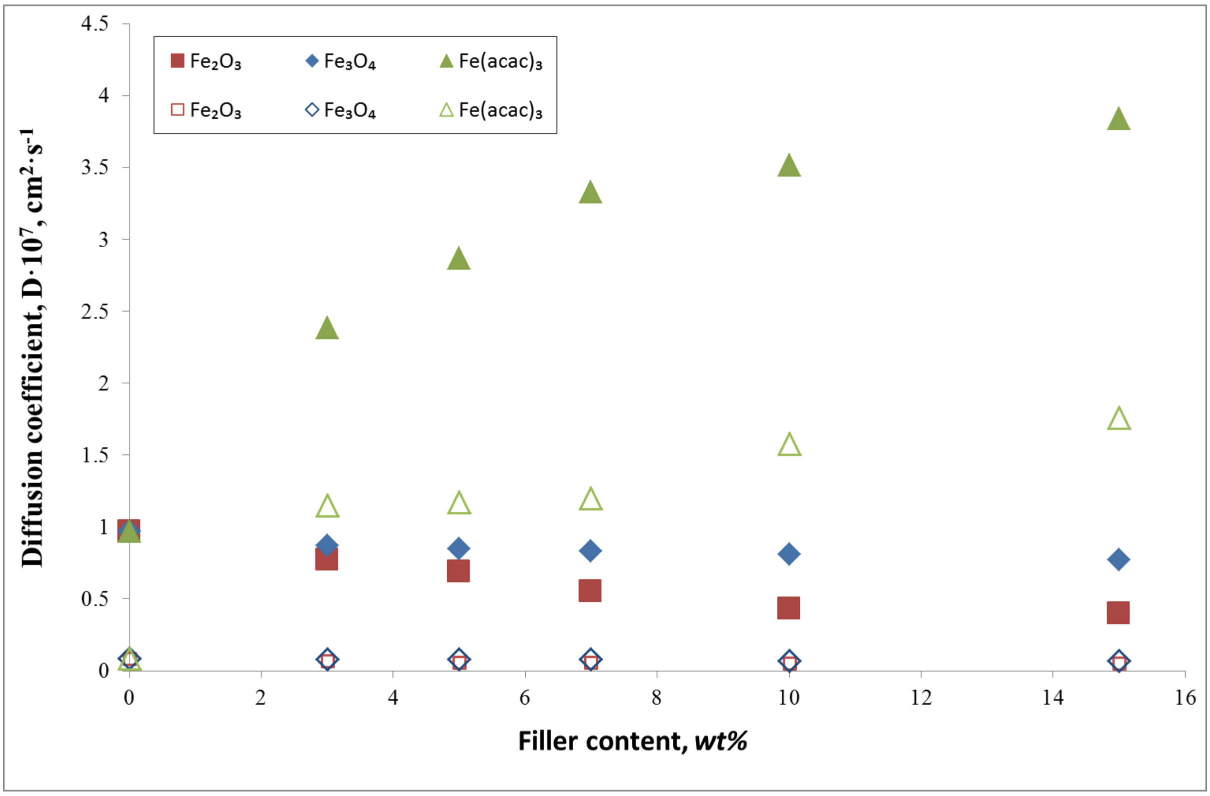

Figure S1. The variation of water (full symbols) and ethanol (blank symbols) diffusion coefficients with increasing content of hematite, magnetite and iron(III) acetylacetonate fillers in ALG membranes.

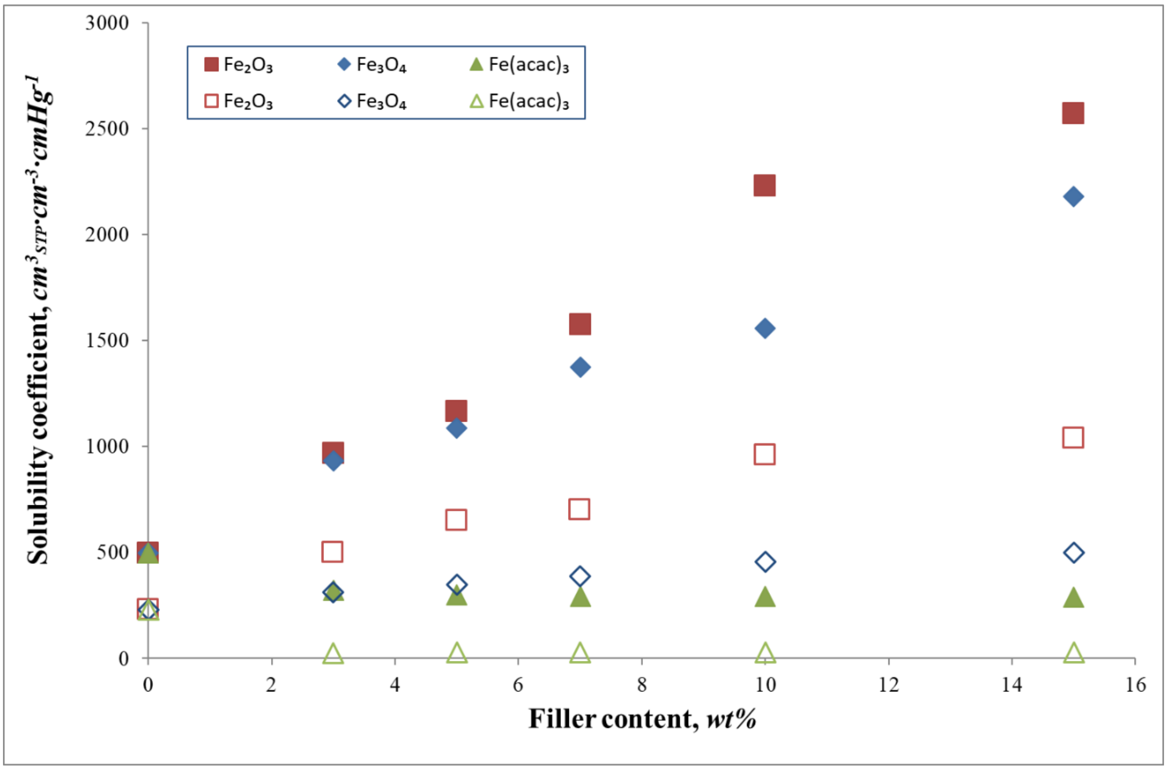

Figure S2. The variation of water (full symbols) and ethanol (blank symbols) solubility coefficients with increasing content of hematite, magnetite and iron(III) acetylacetonate fillers in ALG membranes.

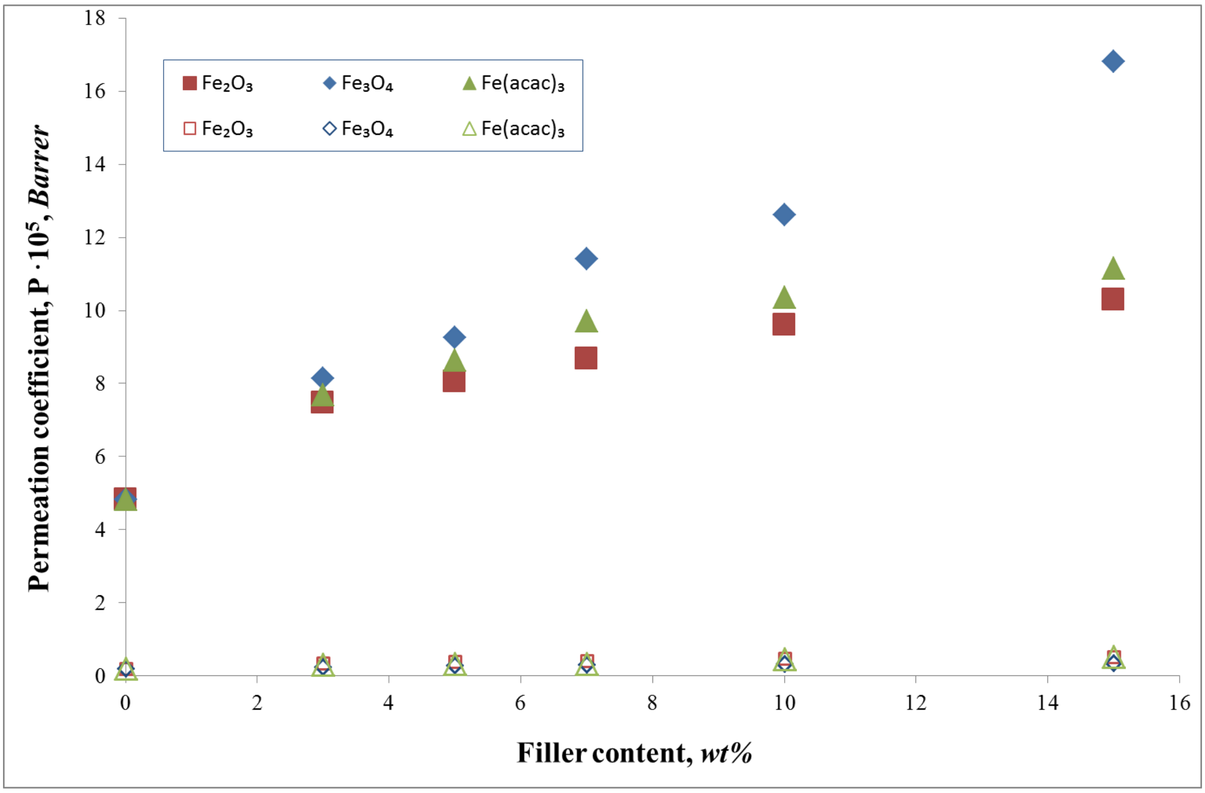

Figure S3. The variation of water (full symbols) and ethanol (blank symbols) permeation coefficients with increasing content of hematite, magnetite and iron(III) acetylacetonate fillers in ALG membranes.

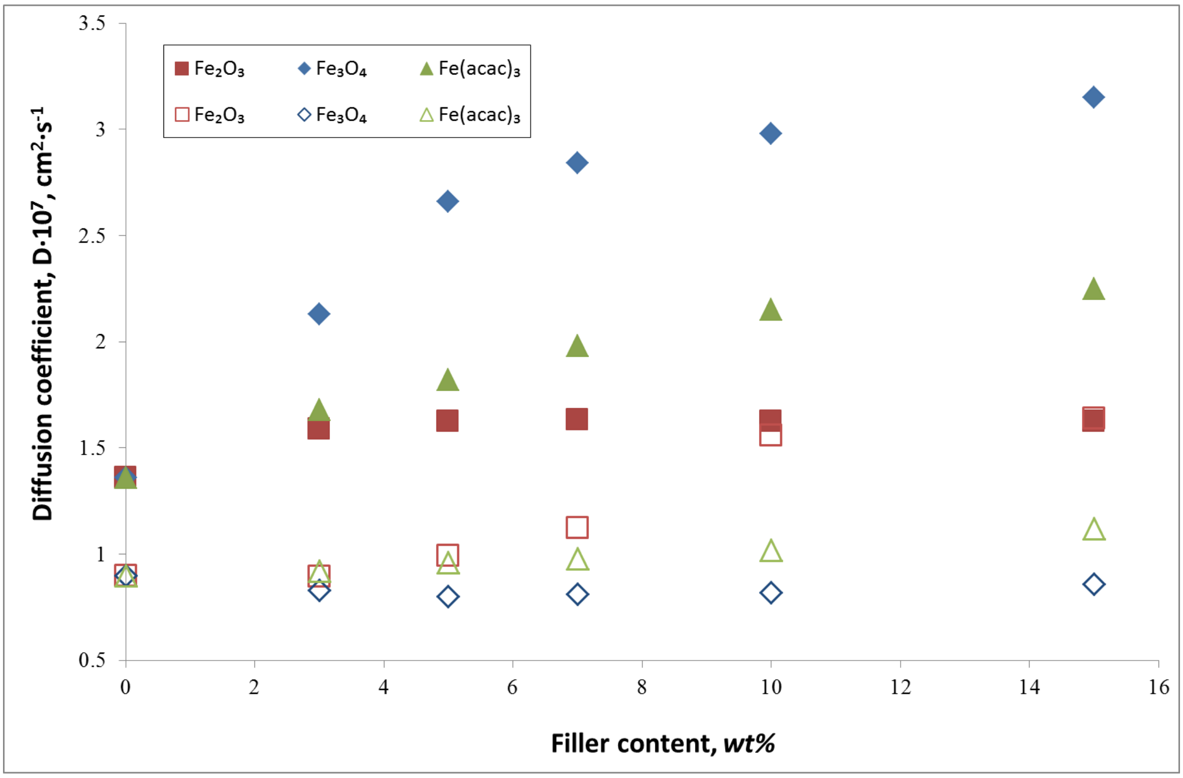

Figure S4. The variation of water (full symbols) and ethanol (blank symbols) diffusion coefficients with increasing content of hematite, magnetite and iron(III) acetylacetonate fillers in PVA membranes.

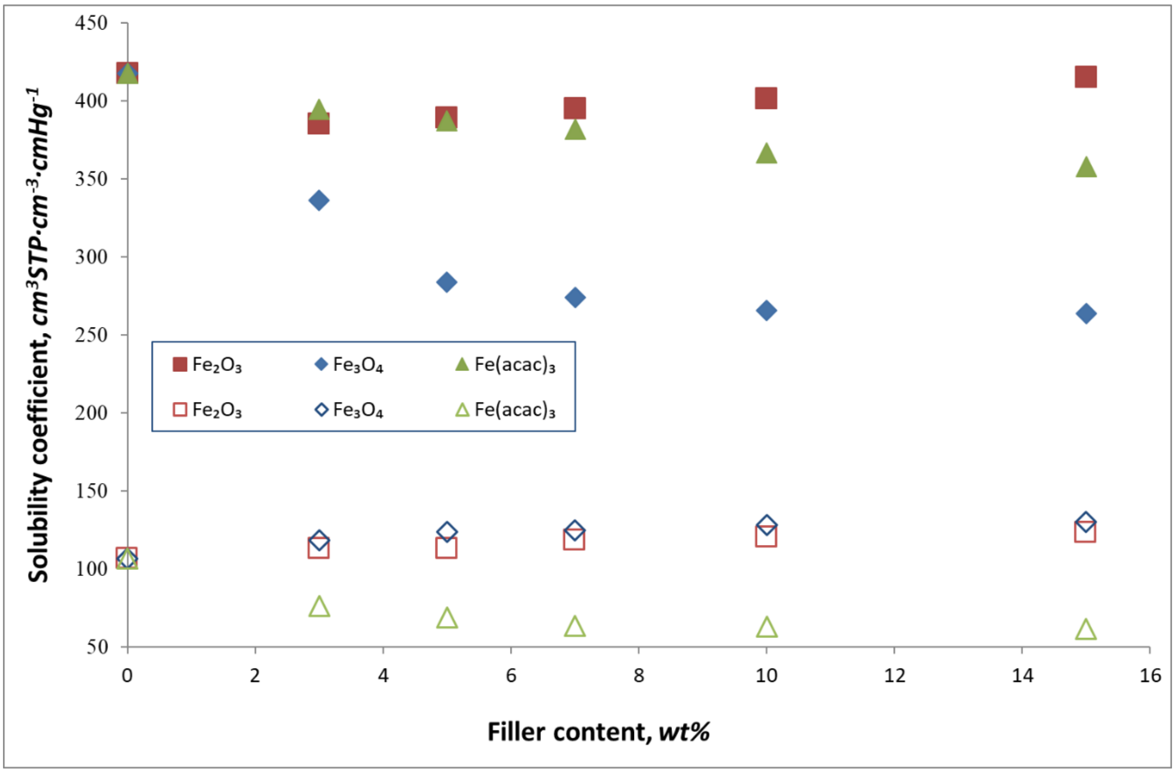

Figure S5. The variation of water (full symbols) and ethanol (blank symbols) solubility coefficients with increasing content of hematite, magnetite and iron(III) acetylacetonate fillers in PVA membranes.

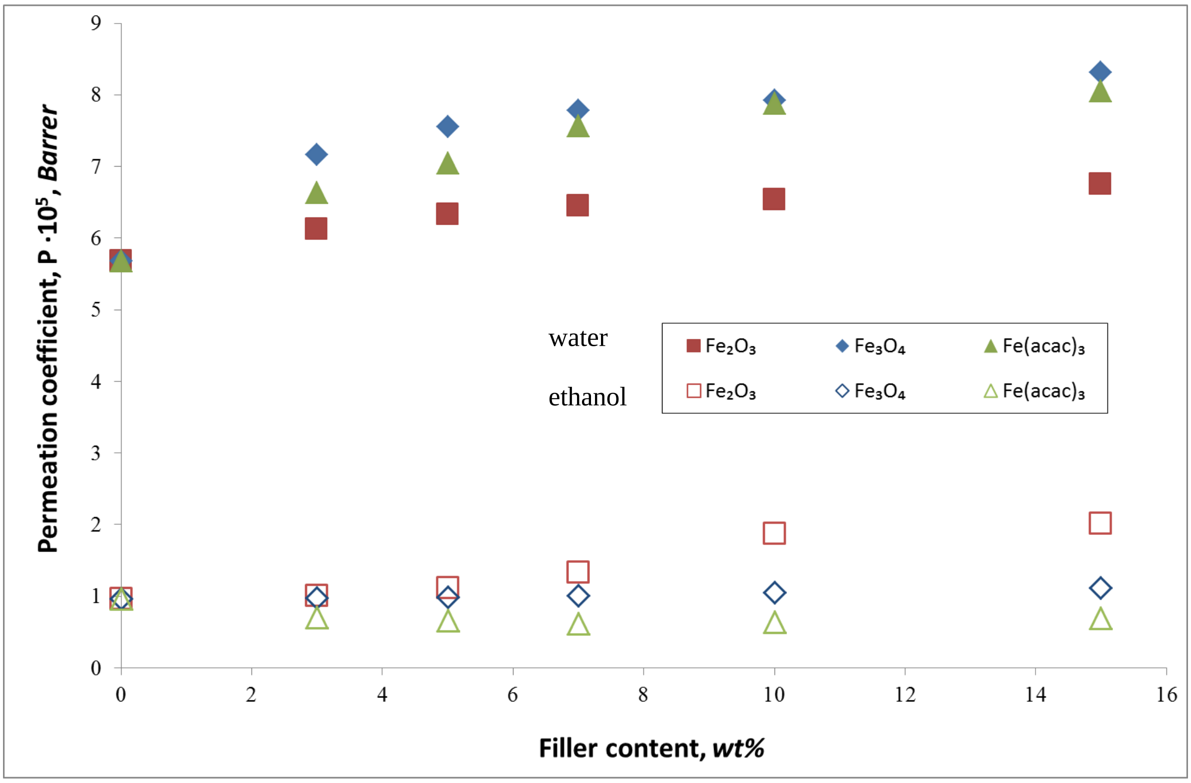

Figure S6. The variation of water (full symbols) and ethanol (blank symbols) permeation coefficients with increasing content of hematite, magnetite and iron(III) acetylacetonate fillers in PVA membranes.
